# A Cysteine Variant at an Allosteric Site Alters MIF Dynamics and Biological Function in Homo- and Heterotrimeric Assemblies

**DOI:** 10.3389/fmolb.2022.783669

**Published:** 2022-02-08

**Authors:** Erin Skeens, Georgios Pantouris, Dilip Shah, Ramu Manjula, Michael J. Ombrello, N. Karl Maluf, Vineet Bhandari, George P. Lisi, Elias J. Lolis

**Affiliations:** ^1^ Department of Molecular Biology, Cell Biology, and Biochemistry, Brown University, Providence, RI, United States; ^2^ Department of Pharmacology, Yale University School of Medicine, New Haven, CT, United States; ^3^ Department of Chemistry, University of the Pacific, Stockton, CA, United States; ^4^ Section of Neonatology, Department of Pediatrics, Cooper University Hospital, Camden, NJ, United States; ^5^ Translational Genetics and Genomic Unit, National Institute of Arthritis and Musculoskeletal and Skin Diseases, Bethesda, MD, United States; ^6^ KBI Biopharma, Louisville, CO, United States

**Keywords:** macrophage migration inhibitory factor, allosteric site, mutagenesis, biophysics, biological activities

## Abstract

Macrophage migration inhibitory factor (MIF) is an inflammatory protein with various non-overlapping functions. It is not only conserved in mammals, but it is found in parasites, fish, and plants. Human MIF is a homotrimer with an enzymatic cavity between two subunits with Pro1 as a catalytic base, activates the receptors CD74, CXCR2, and CXCR4, has functional interactions in the cytosol, and is reported to be a nuclease. There is a solvent channel down its 3-fold axis with a recently identified gating residue as an allosteric site important for regulating, to different extents, the enzymatic activity and CD74 binding and signaling. In this study we explore the consequence of converting the allosteric residue Tyr99 to cysteine (Y99C) and characterize its crystallographic structure, NMR dynamics, stability, CD74 function, and enzymatic activity. In addition to the homotrimeric variant, we develop strategies for expressing and purifying a heterotrimeric variant consisting of mixed wild type and Y99C for characterization of the allosteric site to provide more insight.

## Introduction

Macrophage migration inhibitory factor (MIF) is a pro-inflammatory cytokine involved in multiple non-overlapping physiological pathways (Bernhagen et al., 2007). It is expressed in cells from a variety of tissues with pronounced expression in the cells of the immune system. MIF forms a homotrimeric structure with a solvent channel coincident with its 3-fold axis and possesses three putative catalytic cavities formed at the interface between each of two adjacent subunits (Sun et al., 1996; Suzuki et al., 1996). These were first identified by the similarity of the MIF structure to two microbial enzymes that catalyzed isomerase or tautomerase reactions with a conserved and unusual N-terminal proline functioning as a catalytic base (Stivers et al., 1996a; Stivers et al., 1996b; Subramanya et al., 1996). These enzymes and MIF have 5–20% sequence homology but share an N-terminal proline that is evolutionarily invariant for MIF (Swope et al., 1998). The *bona fide* substrate for MIF has yet to be identified, but “pseudosubstrates” D-dopachrome and hydroxyphenylpyruvate have been useful for designing inhibitors or optimizing high throughput screens (Rosengren et al., 1996; Rosengren et al., 1997; Lubetsky et al., 2002; Cho et al., 2011). These inhibitors were used as tools to probe MIF biological activities and signaling, including functional interactions with p53, ERK-1/2, PI3K/Akt in the cytosol (Hudson et al., 1999; Mitchell et al., 1999; Li et al., 2009), and the extracellular receptors CD74, CXCR2, and CXCR4 (Leng et al., 2003; Bernhagen et al., 2007; Weber et al., 2008; Rajasekaran et al., 2016; Lacy et al., 2018). More recently, a nuclease function was ascribed to MIF in a process known as parthanatos (Wang et al., 2016; Wang et al., 2021).

**FIGURE 1:**
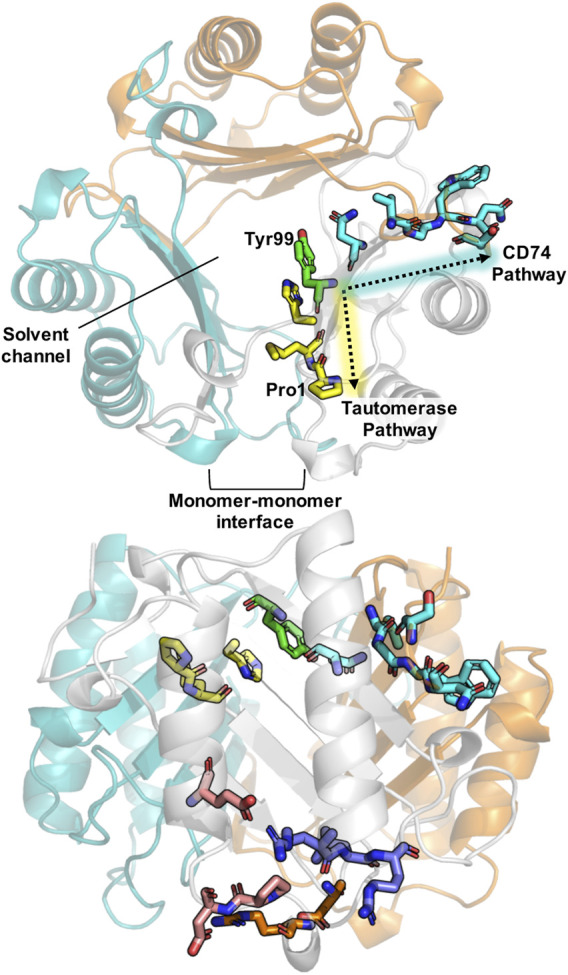
Schematic of important structural features in MIF. A top view of the MIF trimer (top structure) shows the monomeric subunits in gray, teal, and orange (PDB: 1MIF). Previously characterized allosteric pathways controlling tautomerase activity and pro-inflammatory CD74 activation are denoted in yellow and teal, respectively, including the common allosteric node, Tyr99, highlighted in green. Pro-1, the catalytic base for tautomerization, and the monomer-monomer interface, essential for catalysis, are also indicated. A side profile of MIF (bottom structure) highlights the allosteric residues and sites shown above, as well as regions of the protein involved in binding to the receptors CXCR4 (purple) and CXCR2 (orange), and residues implicated in nuclease activity (pink).

The mechanisms by which MIF promotes these activities are unclear, though signaling studies, biochemistry, and structures have provided some molecular details of possible mechanisms. A hypothesis that trimeric MIF functions as an enzyme at higher concentrations in the cytosol and subsequently dissociates into monomers when it is diluted in the extracellular milieu to activate receptors was disproven. Sedimentation velocity and equilibrium ultracentrifugation experiments found MIF has an unusual partial volume for a protein and forms an extremely tight trimer (Philo et al., 2004). Up to four structure-based mutations at the subunit interface also failed to disrupt the homotrimer; MIF with a fifth mutation did not express or was extremely unstable (Fan et al., 2013). Despite the tight homotrimeric association of MIF, it displays remarkable dynamics by NMR (Cho et al., 2010; Crichlow et al., 2012; Pantouris et al., 2018; Pantouris et al., 2020). Recently, we discovered an allosteric regulatory site at the center of the symmetric MIF homotrimer that serves as a hub for two of its biochemical activities: the enzymatic enol-keto tautomerase and CD74 receptor activation (Pantouris et al., 2018; Pantouris et al., 2020) (Figure 1). The allosteric residues, Tyr99 from all three subunits, modulate enzymatic and CD74 receptor activation to different extents when mutated, while concurrently altering the structural and dynamic properties of the protein, as well as hydrogen bonding with solvent molecules in the central solvent channel. Notably, the size of the solvent channel opening and dynamics at the allosteric site were found to be critical diagnostics of functional outcomes, though it is still unclear exactly how the chemical properties of the allosteric site at Tyr99 or the solvent channel influence the biophysical signatures of MIF. Further insights would facilitate the rational design of site-specifically modified allosteric pathways to regulate the information flow to-and-from Tyr99, creating enhanced spatial and temporal resolution of MIF function.

To investigate this relationship further, we introduced a cysteine residue at position 99 (Y99C). The sulfhydryl group of Cys99 is the same molecular distance (∼8 Å) from the enzymatic active site as the Cγ of Tyr99. We hypothesized that this mutation would enable disulfide stabilized dimers within the MIF trimers, providing a more comprehensive understanding of the structural, dynamic, and functional impact of Cys99 substitution. This method was used in a previous MIF study, when a cysteine mutation was introduced at N110 and formed an inter subunit disulfide with a Cys80 and induced changes in the oligomeric and functional properties (Fan et al., 2013). We generated a unique Y99C MIF cysteine variant, as well as a method to generate homo- and hetero-trimeric Cys proteins, and characterized their molecular structure, dynamics, and effect on enzymatic and CD74 activities using X-ray crystallography, NMR spectroscopy, biochemical assays, and *in vivo* experiments.

## Materials and Methods

### MIF Mutagenesis and Protein Purification

The Y99C MIF cDNA was produced by site-directed mutagenesis using the PfuTurbo DNA polymerase (Agilent). DpnI was utilized to digest the parental pET-11b plasmid with the WT MIF cDNA that contained methylated and hemimethylated DNA (Pantouris et al., 2018). BL21-Gold (DE3) cells (Agilent) were transformed with the sequenced Y99C MIF plasmid and selected for on LB agar containing 100 μg/ml Amp (LB/Amp). WT MIF or Y99C MIF-containing cells were grown in 1L LB/Amp at 37°C, and induced by 1 mM of isopropyl β-d-1-thiogalactopyranoside (IPTG) at an OD_600_ of 0.6. Levels of expression were assessed using a NuPAGE 4–12% Bis-Tris gels 4 h after addition of IPTG. The cells were centrifuged and stored in −80°C until further use. Frozen cells were thawed on ice and dissolved in 20 mM Tris, 20 mM NaCl, pH 7.4 containing a mini EDTA-free protease inhibitor cocktail tablet (Sigma-Aldrich) and lysed by sonication. After centrifugation at 4°C, the filtered supernatant was loaded onto Q-Sepharose and SP ion-exchange chromatography columns connected in series. MIF mutant was in the flow-through with ∼95% purity. After concentrating the sample using 10 kDa MWCO Amicon centrifugal filters (Millipore), the protein was loaded onto a 16/60 Superdex 75 size-exclusion column to remove the remaining ∼5% of contaminants with 20 mM Tris, pH 7.4, with 20 mM NaCl as the running buffer. Protein concentration was determined using the Pierce BCA Protein Assay Kit (Thermo Fisher Scientific).

### Y99C/WT MIF Mixed Trimer: Plasmid Design, Expression, and Purification

The Y99C MIF sequence was synthesized and cloned into the pET-24b (+) vector (Kan-resistance) with a C-terminal TEV protease cleavage site, followed by a SNAP-tag (19.4 kDa) and a His_6_-tag (GenScript United States, Inc.). The Y99C MIF/SNAP-tag/His_6_-tag plasmid was co-transformed with the WT MIF plasmid (pET-11b; Amp-resistance) into BL21-Gold (DE3) competent cells (Agilent) and selected for on LB agar plates containing 1.0 mg/ml Amp and 0.75 mg/ml Kan. 1 L LB with 1.0 mg/ml ampicillin and 0.75 mg/ml kanamycin was inoculated with a colony containing the WT MIF and the Y99C MIF/SNAP/His_6_ plasmids, and grown at 37°C to an OD_600_ of 0.6–0.8. Cells were cooled on ice for 30 min, then induced with 0.2 mM IPTG and incubated for 16 h at 18°C. The cells were harvested by centrifugation and stored at −80°C.

Cells were resuspended in 25 ml lysis buffer (20 mM sodium phosphate, 300 mM NaCl, and 5 mM imidazole at pH 7.4) with a mini EDTA-free protease inhibitor cocktail tablet, lysed by sonication, and centrifuged to remove cell debris. Y99C/WT MIF mixed trimers were purified by Ni-NTA affinity chromatography, such that WT MIF homotrimers, containing no His_6_-tags, elute from the column in the flow through, while Y99C/WT MIF mixed trimers containing 1-3 monomers of the Y99C MIF/SNAP-tag/His_6_-tag construct are bound to the Ni-NTA resin (GoldBio) and subsequently washed with 50 ml of 20 mM sodium phosphate, 300 mM NaCl, and 25 mM imidazole buffer at pH 7.4 and eluted with 15 ml of 20 mM sodium phosphate, 300 mM NaCl, and 500 mM imidazole buffer at pH 7.4. To confirm the presence of Y99C/WT MIF mixed trimers, the protein was purified over a HiLoad 26/600 Superdex 200 size exclusion column (GE Healthcare) in 20 mM sodium phosphate, 150 mM sodium chloride, and 1 mM EDTA at pH 7.4. The SNAP-tag and His_6_-tag were then cleaved with TEV protease and subsequently removed via Ni-NTA chromatography.

SDS-PAGE band intensities were analysed using densitometry via ImageJ software (NIH) (Schneider et al., 2012). Briefly, the optical density of each band was determined by the software, with band intensities calculated as the area of the resulting optical density peaks. Each lane was analysed separately due to differing protein concentration per sample. The MIF monomer and MIF monomer + SNAP-tag bands were compared relative to each other and normalized such that the MIF monomer + SNAP-tag band was equal to 1.00.

### Analytical Ultracentrifugation (AUC) of the Y99C MIF Homotrimer Using Sedimentation Velocity

Y99C MIF homotrimer samples were loaded into cells with 2-channel charcoal-epon centerpieces with 12 mm optical pathlength. Samples were analyzed in PBS (from Corning). The reference buffer (PBS) was loaded into the reference channel of each cell to serve as an optical reference blank. The cells were then loaded into an AN-60Ti analytical rotor, which was then placed into a Beckman-Coulter ProteomeLab XL-A analytical ultracentrifuge. After thermal equilibrium was established at 20°C, the samples were scanned at 3,000 RPM at 230 nm to confirm proper cell loading. The rotor was stopped, and then accelerated to the final run speed of 50,000 rpm. Scans were recorded at this rotor speed every ∼4 min for 110 total scans for each sample (∼7.3 h total sedimentation time).

The raw data were analyzed using the SEDFIT software, which implements the *c(s)* method developed by Peter Schuck at the N.I.H(Schuck, 2000). In this approach the raw data scans are analyzed by non-linear least squares to determine the sedimentation coefficient distribution associated with the sample. This method models the influence of diffusion on the data in order to enhance the resolution of the corresponding size distribution. A maximum entropy regularization probability of 0.683 (1 σ) was used, and time-invariant noise was removed. The resultant size distributions were graphed, and the peaks were integrated using OriginLab Origin^®^ version 7.

### CD Spectroscopy

CD spectra and thermal unfolding experiments were acquired on a JASCO J-815 spectropolarimeter equipped with a variable temperature Peltier device and using a 2 mm quartz cuvette. CD spectra were collected at 25°C and denaturation curves were recorded at 218 nm over a temperature range of 20–100°C (293K—373K). *T*_m_ for WT MIF and MIF variants were determined via nonlinear curve fitting using GraphPad Prism.

### Crystallization of Y99C MIF, Structure Determination, and Characterization of Solvent Channel

Using the Formulatrix NT8 robot, 400 nl of 18 mg/ml protein was mixed with 200 nl reservoir solution (2 M ammonium sulfate, 3% 2-propanol, 0.1 M Tris-HCl, pH 7.5) in one drop and set to crystallization at 20°C using a hanging drop vapor diffusion method with a reservoir volume of 100 μL. Crystallization was observed with a RI-1000 Formulatrix Imager, and crystals grew to their maximum size in 30 days. Crystals were then flash-frozen in liquid N_2_ with 15% glycerol which acts as a cryoprotectant during the data collection. X-ray crystallographic data sets were collected at 100 K using a Rigaku-007 Micromax Generator with a PILATUS Dectris 200K Pixel Array Detector and AFC 4-axis goniometer. Diffraction data were integrated and scaled using HKL3000 (Minor et al., 2006). The crystal structure was solved by molecular replacement method using the MIF monomer structure (PDB ID:2OOW) and PHASER (McCoy et al., 2007). The structure solution yielded three monomers of MIF in the asymmetric unit. The refinement of the structure was performed using the module *Phenix.refine* of the *PHENIX* package (Adams et al., 2010). Cycles of refinement and model building were done using REFMAC (Winn et al., 2011) and Coot (Emsley and Cowtan, 2004). The stereochemistry of these crystal structures was assessed with *MOLPROBITY* (Chen et al., 2010). The X-ray data collection, scaling, and refinement statistics are summarized in Supplementary Table S1. Figures of the solvent channels and the minimal radius for WT, Y99C, and Y99A were calculated with MOLE 2.0 (Sehnal et al., 2013) and MoleOnline (Pravda et al., 2018), respectively.

### NMR Spectroscopy

Isotopically labeled MIF samples for NMR studies were grown in 1 L M9 minimal media supplemented with glucose (^12^C_6_H_12_O_6_, natural abundance) and ammonium chloride (^15^NH_4_Cl, Cambridge Isotope Labs) as the sole carbon and nitrogen sources, respectively. The ^15^N-MIF samples were expressed and purified as described above, then dialyzed into 20 mM sodium phosphate, 1 mM EDTA, and 7.5% D_2_O at pH 7.0 and concentrated to 0.5—1.0 mM with a 10 kDa MWCO Amicon centrifugal filter (Millipore).

NMR experiments were carried out on a Bruker Avance NEO 600 MHz spectrometer at 30°C. ^1^H-^15^N TROSY HSQC and relaxation data were collected with the ^1^H and ^15^N carriers set to the water resonance and 120 ppm, respectively. NMR spin relaxation experiments were performed using TROSY-based pulse sequences adapted from Palmer and coworkers (Loria et al., 1999). Longitudinal relaxation rates (*R*_*1*_) were measured with *T*_1_ delays of 20, 60, 100, 200, 600, 800, and 1,200 ms. Transverse relaxation rates (*R*_*2*_) were collected with 1.0 ms spacing between 180° CPMG pulses at total relaxation delays of 1, 2, 4, 8, 10, and 12 ms. The recycle delay in these experiments was 2.5 s. Longitudinal and transverse relaxation rates were extracted by non-linear least squares fitting of the peak heights (major peaks in cases of slow exchange) to a single exponential decay using in-house software. Uncertainties in these rates were determined from replicate spectra. The heteronuclear cross-relaxation rate (^1^H-(^15^N) NOE) was obtained by interleaving pulse sequences with and without proton saturation and calculated from the ratio of peak heights from these experiments. NMR data were processed using NMRPipe (Delaglio et al., 1995) and analysed in Sparky (Lee et al., 2015) along with in-house scripts.

### *In vivo* Neutrophil Recruitment Assays

Wild-type mice of genetic background strain (C57BL6/J) were purchased from the Jackson Laboratory (Bar Harbor, ME) and housed in a pathogen-free animal facility at Cooper University Healthcare. All experiments were done in 10–12 weeks old male mice. Mice were administered a one-time intratracheal instillation of 100 µl of normal saline alone (vehicle) or 10 μg/ml of WT-MIF or Y99C MIF in a normal saline solution. Mice were sacrificed after 6 h of vehicle only or vehicle + experimental agent administration and bronchoalveolar lavage (BAL) fluid was collected. BAL collection was performed by cannulating the trachea with a blunt 22-gauge needle and lavaging the lungs with 800 µl of pre-cooled sterile PBS solution. Total protein concentration in the BAL fluid was measured using the Pierce™ BCA assay kit (Thermo Scientific, Rockford, IL), as previously described (Shah et al., 2019). The animal study protocol was approved by the Institutional Animal Care and Use Committee of Cooper University Healthcare, Camden, NJ.

### Steady-State Kinetics

Michaelis-Menten kinetics were performed as previously described (Pantouris et al., 2020). Briefly, a 100 mM stock of 4-hydroxyphenyl pyruvate (4-HPP) in 0.5 M ammonium acetate, pH 6.2 was prepared by overnight equilibration to generate the predominant keto form of 4-HPP. Enzymatic assays used 96-well plates containing a mixture of 10 μL of the keto form of HPP at concentrations ranging from 0–2 mM and 130 μL borate at a final concentration 0.420 M per well. The reaction was initiated with 50 nM protein. The concentration of the enol, which forms an enol-borate complex, was to measure at 306 nm (ε_306_ = 11,400 M−1 cm−1) every 10 seconds for a total of 1.5 min using a Tecan Infinite M200. Each reaction was conducted in triplicate per protein sample.

## Results

### Biophysical Characterization of Y99C and Y99C/WT MIF Trimers

**FIGURE 2:**
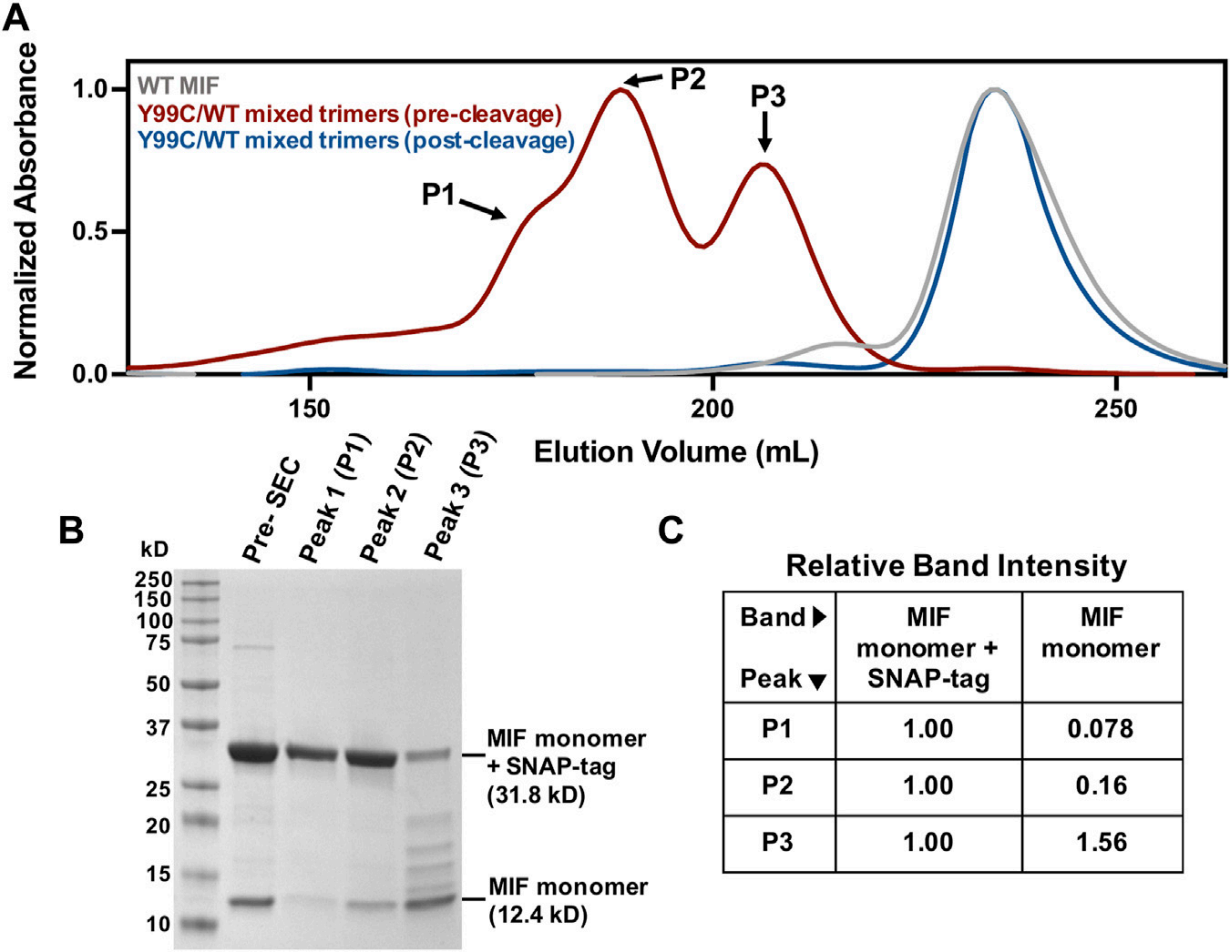
Verification of novel Y99C/WT mixed trimers **(A)** Chromatographic overlay of WT MIF and the Y99C/WT mixed trimers pre- and post-tag cleavage as purified by size exclusion chromatography (SEC) **(B)** SDS-PAGE analysis of the Y99C/WT mixed trimer species showing the presence of the MIF monomer (12.4 kDa) and the MIF monomer + SNAP-tag (31.8 kD) in peaks 1-3 **(C)** Relative SDS-PAGE band intensities of the MIF monomer and the MIF monomer + SNAP-tag bands from **(B)** via ImageJ software (NIH). Each lane was analyzed separately and normalized such that the MIF monomer + SNAP-tag band is equal to 1.00.

Since Tyr99 is located the end of a β-strand of the MIF structure, we speculated it would take local unfolding to move the Cys residues in proximity to form disulfide linkages. We also set out to generate the first mixed trimer species of MIF, with the rationale of using the steric bulk of a Tyr99 side chain to constrain other Cys99 residues into forming disulfide bonds. This approach is also a proof-of-principle demonstration for the engineering of novel MIF variants with structural scaffolds and activities distinct from WT MIF and homotrimeric variants. While the Y99C MIF homotrimer displays a very similar size exclusion chromatographic profile to that of WT MIF, purification of the Y99C/MIF mixed trimers, facilitated by a SNAP-tag and a His_6_-tag on Y99C monomeric subunits, shows distinct MIF species with differing molecular weights that can be followed by SDS-PAGE (Figures 2A,B). The MIF monomer band (12.4 kDa) increases in intensity, relative to the MIF monomer/SNAP-tag band (31.8 kDa), from peak 1 (P1) through peak 3 (P3) on the size exclusion chromatogram, representative of MIF species with increasing numbers of WT MIF monomers within the MIF trimer (Figure 2C). Thus, P1 is likely the Y99C homotrimer (96.1 kDa with SNAP-tags), P2 is the Y99C/WT heterotrimer with two Y99C monomers and one WT monomer (76.7 kDa with SNAP-tags), and P3 is the Y99C/WT heterotrimer with one Y99C monomer and two WT monomers (57.3 kDa with SNAP-tag). When P1-P3 are pooled and cleaved of the SNAP-tags and His_6_-tags, the Y99C/WT mixed trimers elute at the same volume as WT MIF at 37.9 kDa.

The Y99C MIF homotrimer was probed by analytical ultracentrifugation at three loading concentrations (Supplementary Figure S1). The sedimentation coefficient of the Y99C homotrimer is in good agreement with published values for WT MIF (Philo et al., 2004). The molar mass of 37.8 ± 0.8 kDa calculated directly from the primary data agrees very well that of WT MIF (37.9 ± 2.8 g/mol per trimer). Thus, the Y99C variant is a trimer and also has the same elevated partial specific volume seen for WT MIF (within error). With these data, we see no differences between WT MIF and the Y99C MIF homotrimer in terms of oligomeric state, shape, or partial specific volume.

### X-Ray Crystallography of the Y99C MIF Homotrimer

**FIGURE 3:**
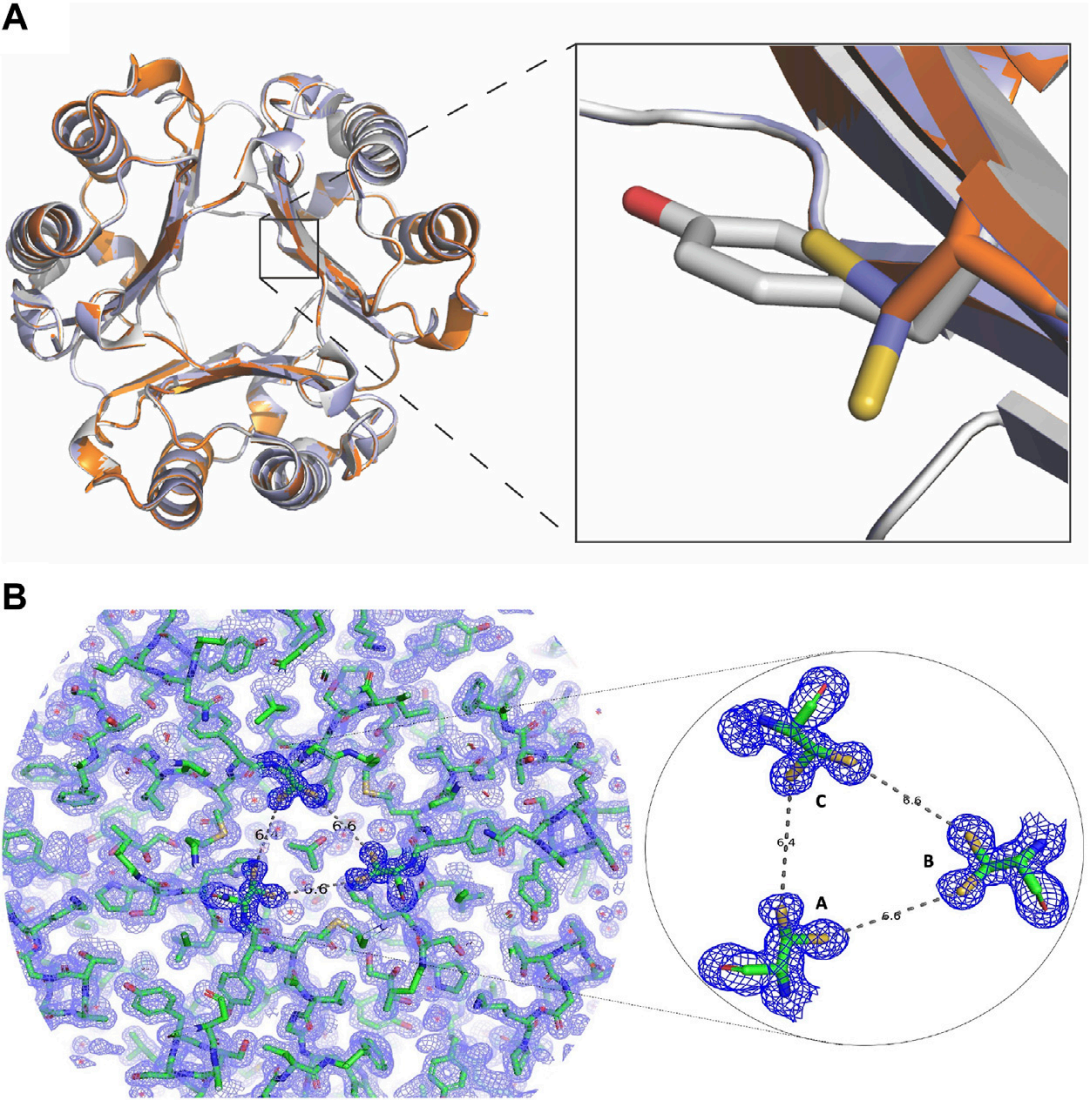
Changes in the MIF structure with mutations at Tyr99 **(A)** The crystal structure of Y99C MIF has two thiol group conformations per monomer in the solvent channel (PDB: 7KQX). The inset shows overlapping side chains for residue 99 in WT MIF (3DJH; gray) and the Y99C (7KQX; blue) and Y99A (5EIZ; orange) MIF variants **(B)** Electron density map for MIF Y99C homotrimer. On the left, the 2Fo-Fc electron density map for the Y99C MIF homotrimer is shown. On the right, the 2Fo-Fc map and the two conformations for the thiol groups of Y99C are enlarged. The distances among the closest sulfur atoms from each subunit are over 6 Å apart and too far from each other to form disulfides.

The structure of homotrimeric Y99C MIF was solved to 1.60 Å to probe whether there were sufficient protein dynamics for the Cys99 side chain to form a disulfide and to detect any unexpected effects. The structural alignment of WT MIF, Y99A, and Y99C were calculated and viewed in PyMol (Figure 3A). From this alignment, the residue 99 sidechains indicate the Cβ carbons overlap very well (Figure 3A, right panel). Interestingly, the thiol group in all three monomers has two conformations in the channel as shown in Figure 3A where Y99 and Y99A are also shown. The 2Fo-Fc and omit map of Y99C is also shown (Figure 3B, Supplementary Figure S2). The closest distances among the thiol groups of adjacent subunits are ∼6.5 Å (Figure 3B). The two conformations of the thiol groups in this study do not interact with other protein atoms nor do they induce large conformational changes (Figure 3C). The RMSD of Y99C to WT MIF (3DJH) and the Y99A (5EIZ) structures are 0.134 Å and 0.077 Å, respectively, and support a lack of sufficient movement of Y99C to induce conformational changes. Though the shared Cβ atom for WT, Y99A, Y99C MIF align very well, the radius of the solvent channel opening where the three Y99C residues appear is 3.4 Å and similar to Y99A (3.6 Å), in contrast to WT MIF (1.4 Å) as calculated by MOLEonline (Pravda et al., 2018). In a previous study involving three Tyr99 variants and four His62 mutations, there was an inverse correlation between the size of the opening and catalytic efficiency (Pantouris et al., 2020).

### The MIF Structure Is Destabilized by Cys99 Residues

To assess the impact of the Cys99 mutation on the secondary structure and stability of MIF, far-UV circular dichroism (CD) experiments were conducted. CD spectra of the Y99C homotrimer and Y99C/WT mixed trimers display α-β secondary structure consistent with that of WT MIF, with only subtle differences observed (Supplementary Figure S3A), which is in good agreement with the crystallographic analysis of the Y99C homotrimer. Similarly, thermal unfolding experiments reveal that the Y99C mutation has a destabilizing effect on the MIF structure, with *T*_m_ values for the Y99C homotrimer and Y99C/MIF mixed trimers of 76.9°C (Δ*T*_m_ = −1.6°C) and 77.5°C (Δ*T*_m_ = -1.0°C), respectively, relative to WT MIF (*T*_*m*_ = 78.5°C). (Supplementary Figure S3B). Together, these data suggest that MIF becomes increasingly destabilized as the number of Cys99 residues increases per trimer.

### NMR Spectroscopy Highlights Altered Solution Structures and Dynamics of Y99C Variants

**FIGURE 4:**
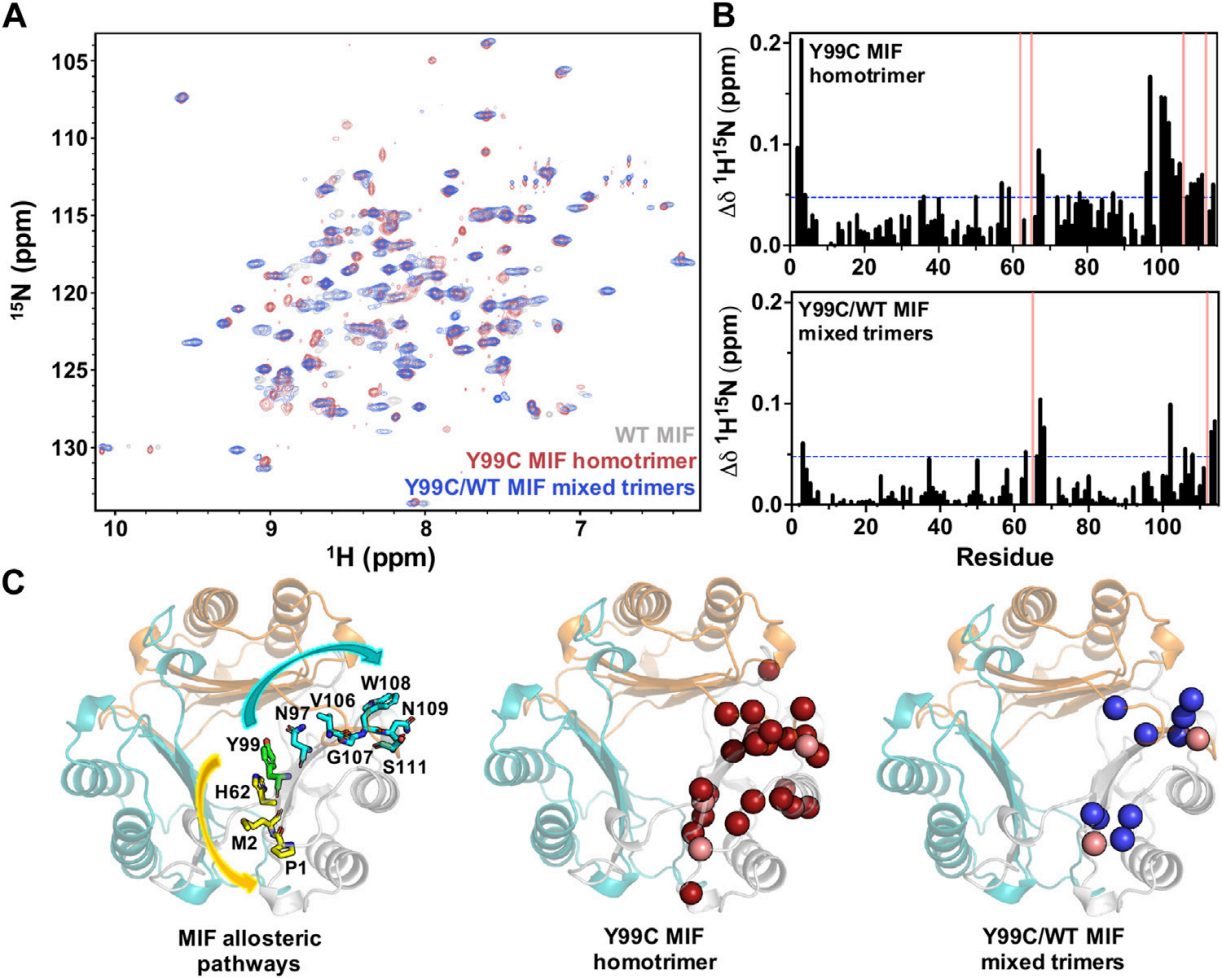
**(A)** ^1^H-^15^N TROSY HSQC spectral overlay of WT MIF (gray), the Y99C homotrimer (red), and the Y99C/WT mixed trimers (blue) **(B)** ^1^H-^15^N combined chemical shift perturbations (Δδ) for the Y99C homotrimer (top) and the Y99C/WT mixed trimers (bottom). Pink bars denote residues that are broadened beyond detection, and blue lines represent 1.5σ above the 10% trimmed mean of all shifts **(C)** Significant chemical shift perturbations observed for the Y99C homotrimer (middle, red spheres) and Y99C/WT mixed trimers (right, blue spheres) are mapped onto the MIF structure (PDB: 1MIF) for comparison to the previously characterized MIF allosteric pathways (left) affecting tautomerase activity (yellow) and CD74 activation (teal). Pink spheres denote residues broadened beyond detection. Residues after position 99 are mapped onto the adjacent monomer, as reported for the CD74 activation site.

We recently employed NMR spectroscopy to characterize significant solution structural and dynamic changes associated with mutations at the Tyr99 allosteric site that are not obvious through crystallographic analysis (Pantouris et al., 2020). Here, we find that the NMR spectrum of the Y99C homotrimer is distinct from that of WT MIF, with significant chemical shift perturbations observed in residues at or near sites previously implicated in the allosteric signaling that controls MIF function (Figure 4). Chemical shift perturbations of residues at the N-terminus and proximal to His62 are consistent with prior studies of Tyr99 variants that confirmed the allosteric pathway (Tyr99-His62-Met2-Pro1) that modulates MIF enzymatic activity (Pantouris et al., 2020). We also observed significant perturbations at the C-terminus, where the CD74 receptor activation site has been mapped (Figure 4) (Pantouris et al., 2015; Pantouris et al., 2018). Comparatively, the NMR spectrum of the Y99C/WT mixed trimers reveals chemical shift perturbations in the same regions of the MIF structure as the Y99C homotrimer, though to a lesser extent. The highly symmetric WT MIF appears as a 114-residue monomer without the cleaved initiating methionine with a molecular weight of 12,388 Da protein by NMR, although it is actually a 37,164 Da trimer. While the heterozygous incorporation of mutations into MIF alters its local chemical environment, it does not perturb the global symmetry of MIF to an extent that renders its NMR spectrum more difficult to interpret (Figure 4A). Although the similarity of the NMR spectra indicate that mixed trimers also do not facilitate disulfide formation, this is the first evidence that heterozygous MIF samples can be studied with molecular detail in solution.

In order to assess the impact of the Y99C mutation on dynamically-driven signaling in MIF, we quantified longitudinal (*R*_1_) and transverse (*R*_2_) relaxation rates and the heteronuclear ^1^H-[^15^N] NOE to report fluctuations in the protein backbone (Supplementary Figure S4). We postulated that the dynamic profile of the Y99C/WT mixed trimers would be “in between” that of WT MIF and the Y99C homotrimer, as was observed structurally by chemical shift perturbations of the Y99C variants (Figure 4B). Interestingly, we observe a greater overall deviation from WT-like dynamics in the Y99C/WT mixed trimers. In fact, there are limited differences in flexibility between WT MIF and the Y99C homotrimer. Only a few sites of significantly altered *R*_*1*_ and *R*_*2*_ relaxation rates for the Y99C homotrimer are observed, including Ile67, proximal to the His62 allosteric site, and Ile96 and Ala104, proximal to the Tyr99 allosteric site and the CD74 binding site (Supplementary Figure S4,5). Further, the ^1^H-[^15^N] NOE for the Y99C homotrimer reveals a slight global elevation relative to WT MIF, suggesting that the Y99C homotrimer is actually less flexible overall than WT MIF. In contrast, there is a large variance in the dynamic profile of the Y99C/WT mixed trimers. Regions with altered *R*_*1*_ and *R*_*2*_ relaxation rates include residues at the monomer-monomer interface (3, 5, 32, 36, 49), residues proximal to the His62 allosteric site (63, 67), and residues near Tyr99 and the CD74 allosteric pathway (95-98, 100 102, 107, 113). The increased flexibility of the Y99C/WT mixed trimers is most apparent in the ^1^H-[^15^N] NOE, where a global depression of NOE values is observed, including significant deviations from WT MIF in functionally relevant regions of the protein similar to those observed by *R*_*1*_ and *R*_*2*_ relaxation rates.

**FIGURE 5:**
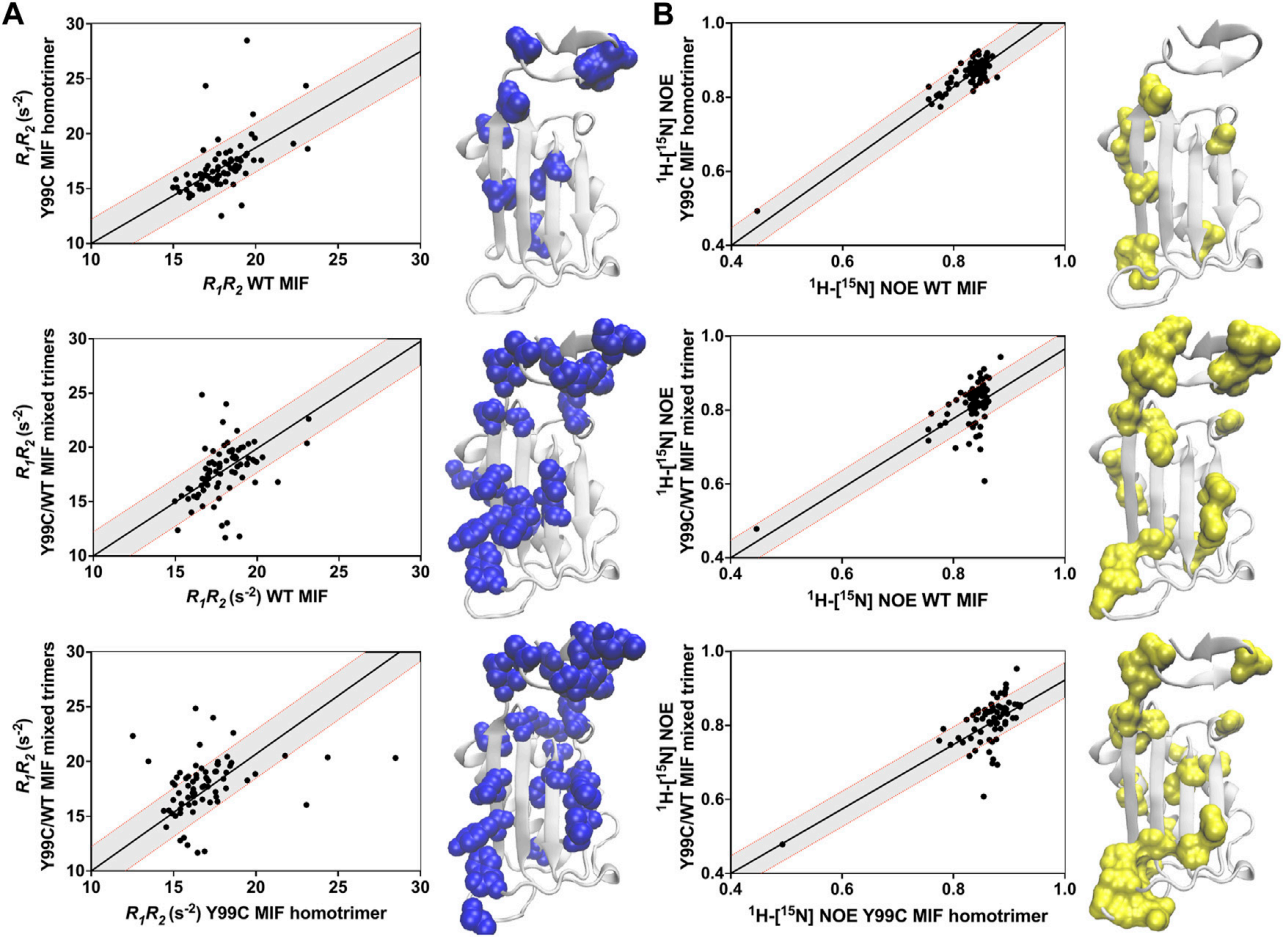
Correlation plots of the **(A)** *R*_*1*_ *R*_*2*_ and **(B)** ^1^H-[^15^N] NOE relaxation rates for the Y99C variants relative to WT MIF and each other. Gray shaded areas denote ±1.5 σ from the linear least-squares fits of the data (black lines). Residues outside of the 1.5 σ correlation boundaries are mapped onto the MIF monomer (PDB: 1MIF).

We compared the dynamics of the Y99C homotrimer and Y99C/WT mixed trimers to those of WT MIF, and to each other, using correlation plots of the *R*_*1*_ *R*_*2*_ relaxation rates (suggestive of μs–ms molecular motions), analyzed as a product to minimize anisotropic molecular tumbling artefacts and ^1^H-[^15^N] NOEs, reporting on ps–ns dynamics (Figure 5). Deviations from linearity in these plots denote substantial differences between the samples, allowing us to pinpoint specific residues affected by disruption of the allosteric site. There is a much lower correlation between the dynamic profiles of WT MIF and the Y99C/WT mixed trimers, indicating that the Y99C/WT mixed trimers exhibit altered flexibility to a much greater extent than the Y99C homotrimers, consistent with our analysis of the raw data. Further, the dynamics of the Y99C variants are distinct from each other, even more so than either variant relative to WT MIF. Residues outside of the 1.5σ correlation boundaries, mapped onto the MIF monomer in Figure 5, highlight regions of altered flexibility suggestive of motion on multiple timescales. Most notably, we observe dynamic changes at the C-terminus and the solvent channel β-sheet, regions that allosterically mediate MIF function (Figure 4C), suggesting that the Y99C/WT mixed trimers may be functionally distinct from WT MIF and the Y99C homotrimers. The heightened flexibility observed for the Y99C/WT mixed trimers may be, in part, due to the heterogeneity of the Y99C/WT mixed trimers, as the subtle loss of symmetry of the heterotrimers may disrupt stabilizing interactions, despite maintaining a relatively similar overall MIF structure by NMR.

### The Y99C Mutation Modulates MIF Enzymatic Activity and Ability to Activate CD74 *in vivo*

The stimulation of neutrophil recruitment in murine lungs is tied to CD74 receptor activation *in vivo* (Takahashi et al., 2009)*.* Our previous work with a Y99A variant showed significant abrogation of neutrophil recruiting activity, suggesting a coupling of this allosteric residue to the C-terminal CD74 binding site (Pantouris et al., 2018). We measured the percentage of neutrophils and total protein content as biomarkers for CD74 activity and alveolar-capillary leak/pulmonary edema, respectively, in bronchoalveolar lavage (BAL) fluid obtained from mice sacrificed after 6 h of administration of a vehicle (saline) only or vehicle with Y99C MIF. The neutrophil influx (Supplementary Figure S5) was significantly increased in the WT MIF group (compared to controls), but significantly decreased in groups administered the Y99C homotrimeric variant, similar to Y99A (Pantouris et al., 2018). The decrease in lung inflammation is likely due to the same factor as the Y99A variant; the absence of Tyr99 and the inability of the variant to undergo correlated motions with a number of proximal and distal residues originally shown by molecular simulations (Pantouris et al., 2018). NMR studies of Y99C (Figure 4A,B, Figure 5) show perturbation in residues Ile64, Lys66, Trp108, and Asn109, which are involved in CD74 receptor binding and activation. We also cannot eliminate the two sidechain orientations of Cys99, the local changes it causes at receptor binding residues, and other residues in its proximity.

We previously noted that increasing the radius of the solvent channel opening by substituting Tyr99 with other residues is correlated to reduced catalytic activity (Pantouris et al., 2020). Though we observed a solvent channel opening for Y99C similar to that of Y99A, the catalytic activity of the Y99C homotrimers has reduced (55%) catalytic efficiency relative to WT MIF and compared to Y99A (77%) (Pantouris et al., 2020) (Supplementary Figure S6). Our previous work evaluated a range of amino acid substitutions at position 99, including glycine, alanine, and phenylalanine. While the catalytic efficiencies of Y99G and Y99F were found to be 10 and 143%, respectively, compared to WT MIF, Y99A only exhibited a small decrease in catalytic activity to 77%, suggesting that amino acids with side chain sizes between the extremes of glycine and phenylalanine are less functionally predictable, and there are likely additional factors that influence activity. The catalytic efficiency of the Y99C/WT mixed trimers was predicted to be between that of the Y99C homotrimer and WT MIF, as there are fewer changes (mutations) compared to the Y99C homotrimer. Interestingly, the catalytic efficiency of the Y99C/WT mixed trimers is reduced to ∼28% of WT MIF. This result may be attributed to the significant increase in flexibility observed by NMR for the Y99C/WT mixed trimers, particularly at the C-terminus near the monomer-monomer interface and in proximity to the allosteric sites on the solvent channel β-sheet, where signaling, ligand binding, and/or active site chemistry may be altered (Supplementary Figure S4,5). We observed limited differences in flexibility of WT MIF and the Y99C homotrimer, which may be a contributing factor in the higher activity of the Y99C homotrimer compared to the heterotrimeric variants. In addition to the altered dynamic profile of the Y99C/WT mixed trimers, the reduced enzymatic efficiency could be due to do the importance of symmetry at the allosteric site and its effects in catalytic activity. The use of heterotrimers was thus important to suggest symmetry is also significant at the allosteric site.

## Discussion

The unexpected discovery of an allosteric site at the center of the MIF trimer has spawned protein engineering and small molecule studies aimed at modulating two non-overlapping functions of MIF from a single location. This approach highlights a strategy to make the catalytic activity, CD74 agonism and antagonism, and conformational ensemble of MIF more intuitively controllable. Mutagenesis studies employed here attempted to probe dynamics at the gating residue of the solvent channel. Engineering of an intersubunit disulfide via mutagenesis at a different residue (N110C) was used to study MIF aggregation by the compound ebselen via interaction with a natural MIF cysteine (Cys80) and the effect of a locked trimer on the catalytic and CD74 activity of MIF (Ouertatani-Sakouhi et al., 2010; Fan et al., 2013). The intersubunit disulfide induced global conformational changes, a higher-order oligomer, and decreased catalytic and CD74 activation (Fan et al., 2013). The X-ray structure of the Y99C MIF homotrimer did not reveal the presence of a disulfide between any two of three subunits. Interestingly, it did reveal two conformations for each thiol group (per monomer). The Y99C homotrimer abolished CD74 activation *in vivo* but possessed reduced enzymatic activity, supporting the role of Tyr99 as a common allosteric node within two distinct allosteric pathways. We also demonstrate in this study a proof-of-principle that MIF can be engineered to form Y99C/WT MIF heterotrimers with different structural and biophysical properties than WT MIF or the corresponding Y99C homotrimeric variant. Surprisingly, the catalytic efficiency of the mixed heterotrimeric variants was lower than Y99C. While NMR studies reveal only slight differences in the dynamics of the Y99C homotrimer and WT MIF, the Y99C/WT mixed trimers studied here exhibit heightened flexibility at critical regions of the protein, as well as the absence of symmetry compared to the Y99C homotrimer, suggesting that the slight loss in symmetry of the heterotrimers may play an important role in the altered dynamics and functional effects of the Y99C/WT mixed trimers. Future studies will attempt to isolate the heterotrimers to study their structures and dynamics individually.

The advance in engineering heterotrimeric MIF species will allow us to characterize the biological activities and biophysical properties of this and other heterotrimeric mutants to provide insight into how this protein carries out its multi-functional activities. It is noteworthy that two other examples of heterotrimeric proteins exist for use as an anti-inflammatory, and in the other case, as a causative agent for inflammatory disease. A strategy of structure-based design was used to convert the inflammatory homotrimeric tumor necrosis factor (TNF) protein into a heterotrimeric mutant that inactivates the native TNF via its sequestration from its two receptors (Steed et al., 2003). A disease-causing example involves more than 100 autosomal dominant mutations in the extracellular binding domain of the heterotrimeric tumor necrosis factor receptor 1 (TNFR1) responsible for the TNF Receptor-Associated Periodic Syndrome (TRAPS). The underlying symptoms are recurrent and prolonged episodes of fever and inflammation (Cudrici et al., 2020). The TNFR2-Fc therapeutic, etanercept, has limited efficacy toward TRAPS, demonstrating that the interactions between TNF and its receptors is not the basis of the disease. This supports the reduced binding of TNF to the TNFR1 heterotrimeric T50M variant and absence of almost any binding to the C33Y and C52F mutants, even though these receptor mutants are present on the cell surface (Todd et al., 2004). Studies initially indicated that variant-containing TNFR1 heterotrimers were mostly retained within the ER, resulting in ER stress in the context of inflammatory signaling and secretion of pro-inflammatory cytokines (Simon et al. 2010). Unexpectedly, the inflammation of TRAPS was not driven by the traditional unfolded protein response, but rather was mediated by enhanced generation of mitochondrial ROS (Simon et al., 2010; Bulua et al., 2011). Our studies of Y99 MIF variants serve as another example of a homotrimeric and heterotrimeric protein with unique biological effects. Heterotrimeric MIF species also provide an additional avenue for characterizing the allosteric network proposed to control the distinct functions of MIF, including the role of inter- and intra-subunit signaling.

## Data Availability

The datasets presented in this study can be found in the online repository BMRB, https://bmrb.io/. The names of the accession number(s) are as follows: BMRB entry assigned accession number: 51223 Title: “1H15N HSQC chemical shifts and Relaxation Parameters for Y99C MIF Homotrimers.” BMRB entry assigned accession number: 51224 Title: “1H15N HSQC chemical shifts and Relaxation Parameters for Y99C MIF Mixed Trimers.”
