# Supplementary material for: A Cysteine Variant at an Allosteric Site Alters MIF Dynamics and Biological Function in Homo- and Heterotrimeric Assemblies
Source: Front Mol Biosci. 2022 Feb 8;9:783669. doi: 10.3389/fmolb.2022.783669 (PMC8893199; doi:10.3389/fmolb.2022.783669)
Supplement: Supplementary file 1 [file DataSheet1.pdf]

Supporting Information

A cysteine variant at an allosteric site in MIF alters protein dynamics and biological function in homo- and hetero-trimeric assemblies

Erin Skeens,[1‡] Georgios Pantouris,[2,3‡] Dilip Shah,[4] Ramu Manjula,[2] Michael J. Ombrello,[5] N. Karl Maluf,[6] Vineet Bhandari,[4] George P. Lisi,[1,*] and Elias J. Lolis[2,*]

[1]Department of Molecular Biology, Cell Biology, & Biochemistry, Brown University, Providence, RI 02903
[2]Department of Pharmacology, Yale University School of Medicine, New Haven, CT 06511
[3]Department of Chemistry, University of the Pacific, Stockton, CA 95211
[4]Section of Neonatology, Department of Pediatrics, Cooper University Hospital, Camden, NJ 08103
[5]Translational Genetics and Genomic Unit, National Institute of Arthritis and Musculoskeletal and Skin Diseases, Bethesda, MD 20892
[6]KBI Biopharma, Louisville, CO 80027

‡These authors contributed equally

*Correspondence: george_lisi@brown.edu, elias.lolis@yale.edu

Table S1. Crystallographic statistics for the Y99C MIF homotrimer

Table S2. Hydroxyphenylpyruvate Tautomerase Activity Kinetic Values

Figure S1. Oligomeric state of the Y99C MIF homotrimer by analytical ultracentrifugation

Figure S2. Omit map for the variant residue Y99C

Figure S3. Secondary structure and stability of the Y99C MIF variants

Figure S4. NMR spin relaxation data of the Y99C MIF variants

Figure S5. *In vivo* CD74 activation of the Y99C MIF homotrimer

Figure S6. Altered HPP tautomerase activity of the WT and Y99C MIF variants

Table S1. Crystallographic Statistics for the Y99C MIF homotrimer (PDB: 7KQX).

	Y99C (7KQX)
**Wavelength** (Å)	1.5428
**Resolution range** (Å)*	48.1 - 1.6 (1.657 - 1.6)
**Space group**	P 21 21 21
**Unit cell dimensions** (Å)	67.984, 68.062, 86.737
**Total reflections**	2201046
**Unique reflections***	53570 (5219)
**Multiplicity***	5.7 (3.4)
**Completeness (%)***	99.55 (98.32)
**Mean I/sigma (I)***	31.40 (3.29)
**Wilson B-factor** ($Å^2$)	14.29
**R-meas***	0.050 (0.391)
**R-pim***	0.018 (0.204)
**CC1/2***	0.997 (0.898)
**Reflections used in refinement***	53547 (5219)
**Reflections used for R-free***	2579 (248)
**R-work***	0.1529 (0.1873)
**R-free***	0.1798 (0.2408)
**Number of non-hydrogen atoms**	3113
**macromolecules**	2622
**ligands**	125
**solvent**	366
**Protein residues**	342
**RMS(bonds)** (Å)	0.016
**RMS(angles)** (°)	1.35
**Ramachandran favored (%)**	98.81
**Ramachandran allowed (%)**	1.19
**Ramachandran outliers (%)**	0.00
**Rotamer outliers (%)**	1.01
**Clashscore**	5.57
**Average B-factor** ($Å^2$)	17.78
**Macromolecules** ($Å^2$)	15.02
**Ligands** ($Å^2$)	32.63
**Solvent** ($Å^2$)	32.52

Table S2. Hydroxyphenylpyruvate Tautomerase Activity Kinetic Values

	Vmax (μM/s)	kcat (s^{-1})	Km (mM)	kcat/Km ($mM^{-1}s^{-1}$)
WT	0.30 ± 0.03	6.05 ± 0.67	2.13 ± 0.35	2.84 ± 0.44
Y99C	0.28 ± 0.04	5.6 ± 0.81	3.57 ± 0.33	1.57 ± 0.17
Y99C-WT MIF mixed trimers	0.13 ± 0.03	2.65 ± 0.61	3.33 ± 0.84	0.80 ± 0.47

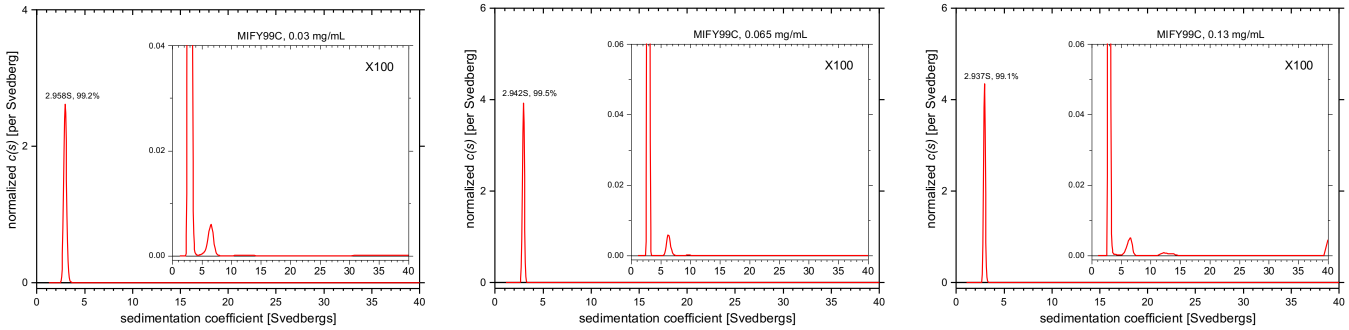

Figure S1. Sedimentation coefficient distribution for the Y99C MIF homotrimer at different concentrations. The inset has the vertical scale expanded 100-fold so that the minor components can be observed. The distribution is normalized so the total area under the curve is 1.0, and thus the area of each peak gives the fraction of that concentration.

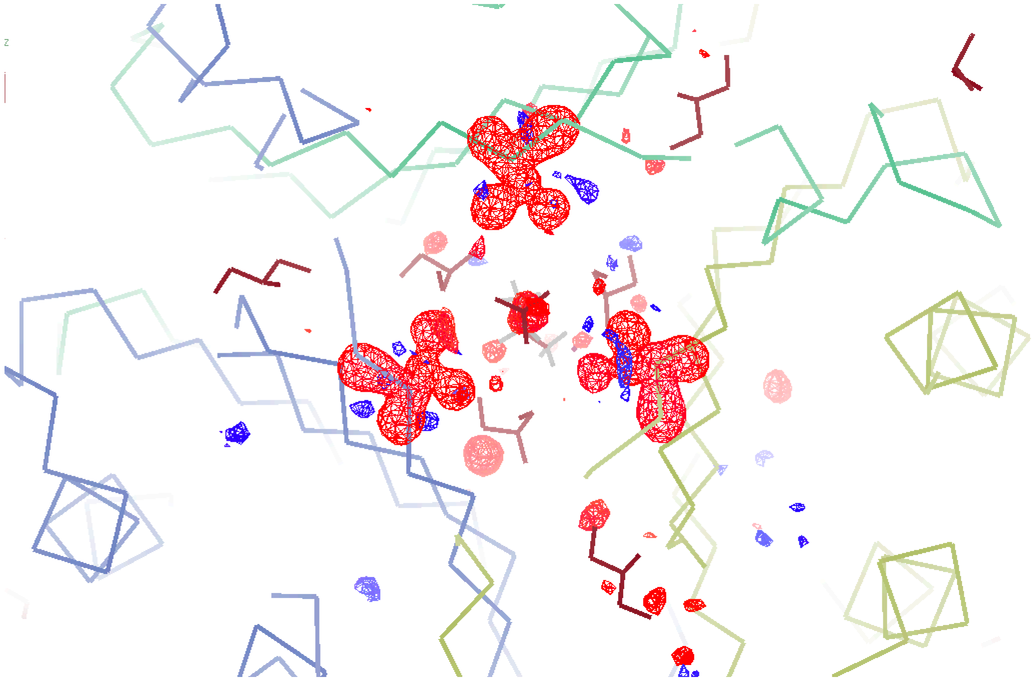

Figure S2. Omit map for the variant residue Y99C from all three subunits in the context of the Cα atoms.

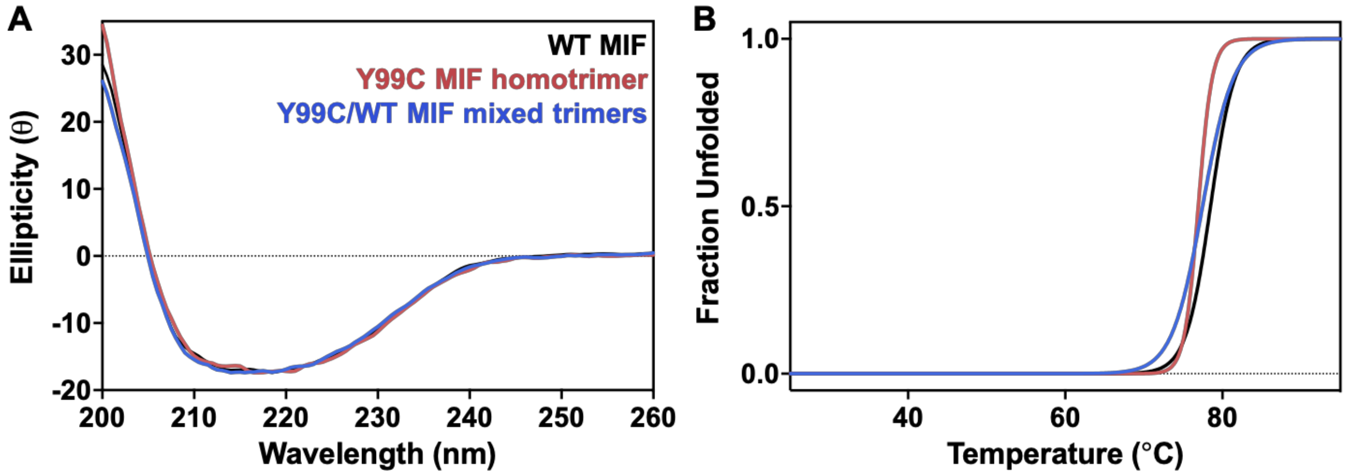

Figure S3. Secondary structure and stability of Y99C MIF variants. **(A)** Far-UV circular dichroism spectra of WT MIF (black), the Y99C MIF homotrimer (red), and the Y99C/WT MIF mixed trimers (blue) at 25°C. **(B)** Thermal stabilities of WT MIF (black; T_m = 78.5°C), the Y99C MIF homotrimer (red; T_m = 76.9°C), and the Y99C/WT MIF mixed trimers (blue; T_m = 77.5°C), normalized and plotted as a fraction of unfolded protein.

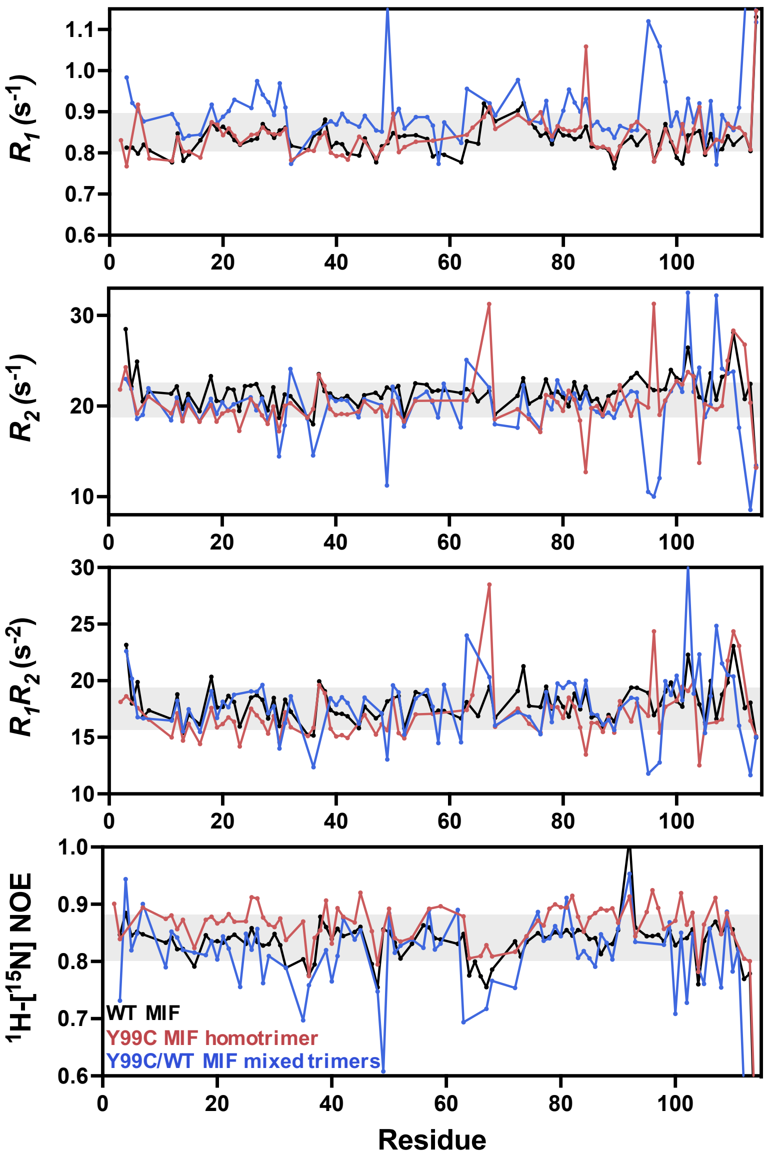

Figure S4. NMR spin relaxation plots of R_1, R_2, R_1R_2, and the ^{1}H-[^{15}N] NOE for WT MIF (black), the Y99C MIF homotrimer (red), and the Y99C/WT MIF mixed trimers (blue). Gray shaded bars represent $\pm1.5\sigma$ of the 10% trimmed mean of all data collected for each relaxation parameter.

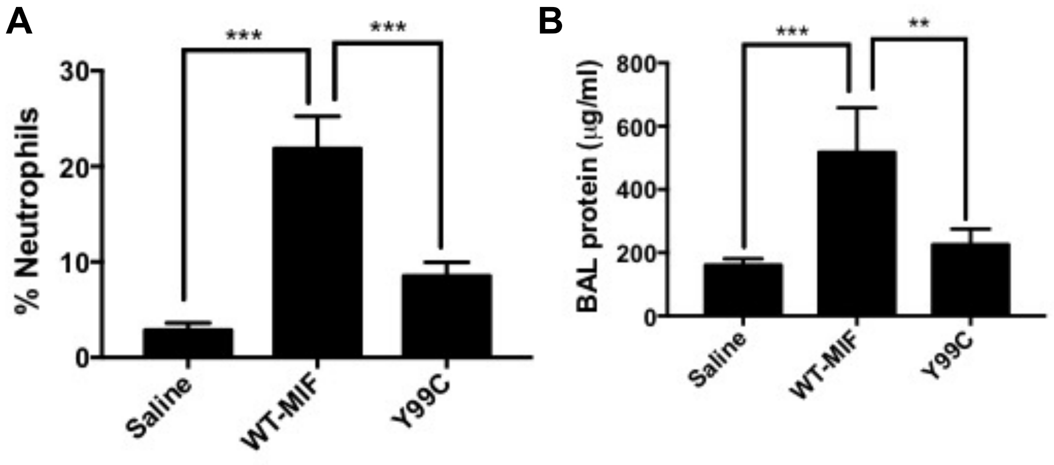

Saline	WT-MIF	Y99C
3.216	25.22	10.52
3.188	19.43	7.81
3.299	18.5	8.56
1.732	24.25	7.25

Saline	WT-MIF	Y99C
154.6397	703.2055	244.7763
189.1451	544.7622	156.0481
157.4565	439.1381	232.805
142.6684	384.2064	269.423

Figure S5. *In vivo* CD74 activation in the lung by WT MIF and the Y99C MIF homotrimer. **(A)** Neutrophil recruitment demonstrates a statistically significant decrease in cells recruited to the lung by the Y99C MIF homotrimer compared to that of WT MIF. **(B)** The Y99C MIF homotrimer leads to significantly decreased total BAL protein levels, a surrogate marker for alveolar–capillary leak/pulmonary edema *in vivo* . $**p < 0.01$, $***p \leq 0.001$.

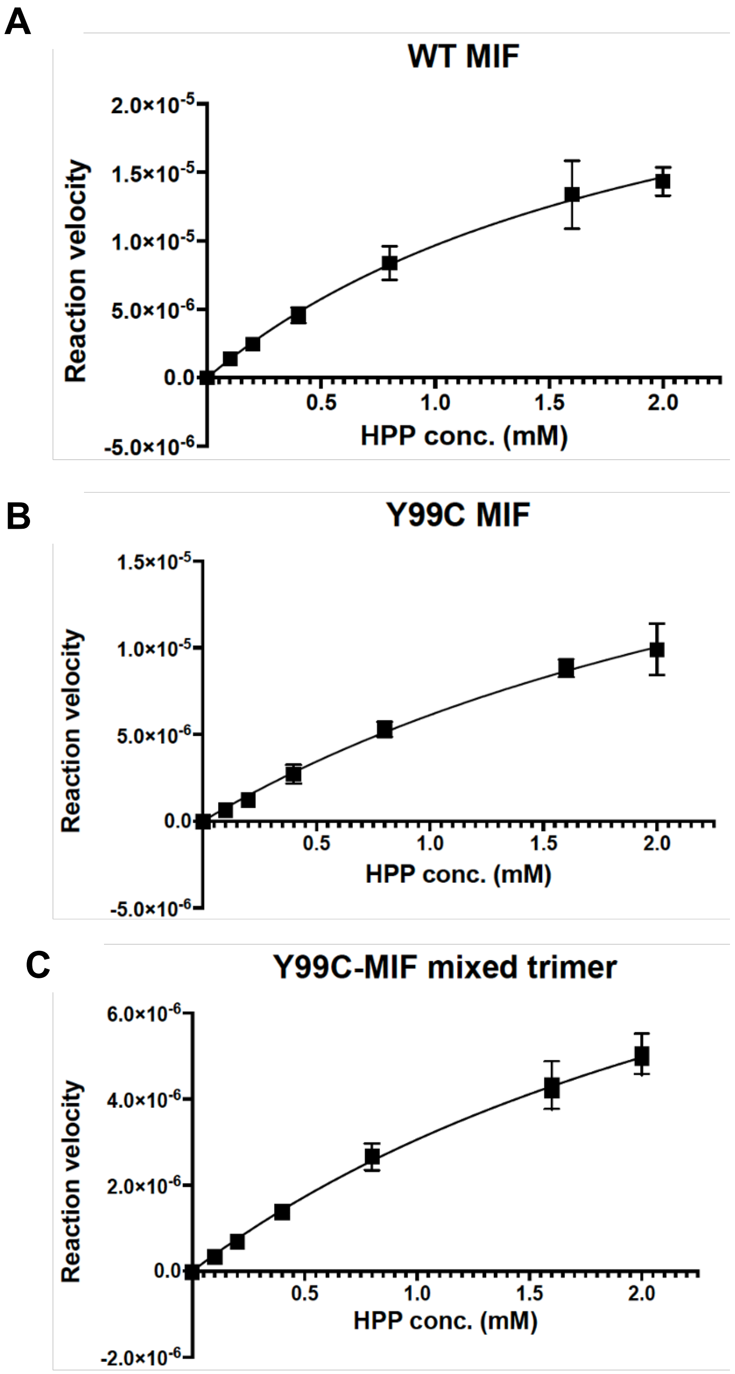

Figure S6. Altered HPP tautomerase enzymatic effiency of the Y99C MIF variants. Hydroxyphenylpyruvate keto-enol tautomerase activity of **(A)** wild-type, **(B)** Y99C mutant, **(C)** and Y99C-WT MIF heterotrimer mixture was monitored by the formation of the HPP keto-enol complex

at λ_{max}= 306 nm with 50 nM MIF and 0-2mM of HPP. Reactions were repeated in triplicate per protein sample, with black box representing the mean and lines representing the standard deviation.
